# Clinical Profile and HLA Typing of Autoimmune Hepatitis From Pakistan

**DOI:** 10.5812/hepatmon.13598

**Published:** 2013-12-16

**Authors:** Nasir Hassan, Adeelur Rehman Siddiqui, Zaigham Abbas, Syed Mujahid Hassan, Ghous Bux Soomro, Muhammed Mubarak, Sabiha Anis, Rana Muzaffar, Mirza Naqi Zafar

**Affiliations:** 1 Department of Hepatogastroenterology, Sindh Institute of Urology and Transplantation, Karachi, Pakistan; 2 Department of Pathology, Sindh Institute of Urology and Transplantation, Karachi, Pakistan; 3 Molecular Diagnostics and Immunology, Sindh Institute of Urology and Transplantation, Karachi, Pakistan

**Keywords:** Hepatitis, Autoimmune, Histocompatibility Testing, Alleles, HLA-DR6 Antigen, Pakistan

## Abstract

**Background:**

Human leukocyte antigen (HLA) typing in autoimmune hepatitis (AIH) has been investigated in different populations and ethnic groups, but no such data is available from Pakistan.

**Objectives:**

The aim of this study was to evaluate the clinical profile of autoimmune hepatitis (AIH), and determine the associated antigens and alleles by performing HLA typing.

**Patients and Methods:**

A total of 58 patients, diagnosed and treated as AIH in the last 10 years were reviewed. Diagnosis was based on International AIH Group criteria. Forty one patients underwent liver biopsy. HLA typing was performed in 44 patients and 912 controls by serological method for HLA A and B, and by PCR technique using sequence specific primers for DR alleles.

**Results:**

Of 58 cases, 35 were females (60.3%). The median age was 14.5 (range 4-70 years), and AIH score was 14 (10-22). Thirty-six (62.0%) patients had type 1 AIH, 10 (17.2%) type 2, and the remaining 12 were seronegative with biopsy proven AIH. Forty-nine patients (84.4%) had cirrhosis. Twenty-four (41.4%) patients had ascites at the time of presentation. Among 41 patients who underwent liver biopsy, thirty-two had advance stages III and IV disease, and twenty had severe grade of inflammation. Fifteen patients had other associated autoimmune diseases and one developed hepatocellular carcinoma. HLA A2 (P = 0.036), HLA A9 (23) (P = 0.018), HLA A10 (25) (P = 0.000), HLA A19 (33) (P = 0.000), HLA B15 (63) (P = 0.007), HLA B40 (61) ( P = 0.002), HLA DR6 (P = 0.001) with its subtypes HLA-DRB1*13 (P = 0.032) and HLA-DRB1*14 (p = 0.017) were more prevalent in AIH with statistical significance than controls.

**Conclusions:**

AIH in our region presents with advanced disease affecting predominantly children and adolescents. There is a genetic association of HLA DR6 along with other alleles and antigens in our patients with AIH.

## 1. Background

Autoimmune hepatitis (AIH) is a less common cause of chronic liver disease (CLD), with low prevalence in Asian countries (1-3). Its pathophysiology remains obscure but is assumed to be related to an autoimmune process (4, 5). Hereditary tendency combined with environmental factors lead to a breach in immune tolerance mechanisms which provoke a T cell mediated immune injury targeting hepatocyte antigens (6, 7). It is more common in females. Diagnosis of AIH is made in the presence of raised gamma globulin levels, autoimmune serology, characteristic features on histopathology, and response to immunosuppressive treatment. One of the reasons of apparent low prevalence of AIH is thought to be lack of knowledge and awareness among clinicians, especially when viral hepatitis is the diagnosis in most patients (8).

HLA typing in AIH has been extensively investigated in the past two decades, and its association in different populations and ethnic groups has been published before (9). Varied associations identified in different ethnic groups emphasize the importance and significance of studying ethnically homogeneous group of patients. No such data is available from Pakistan.

## 2. Objectives

The aim of this study was to perform and evaluate the clinical profile of AIH, and determine its associated HLA alleles by performing tissue typing. This is the first study to address this issue from our country.

## 3. Patients and Methods

A total of 58 patients, diagnosed as AIH at our department in the last 10 years were included in this study; these cases were diagnosed on the basis of International AIH Group criteria using scoring calculator (10-15). A score of > 15 was taken as confirmed AIH, and ≥ 10 as probable AIH. Patients with a score of less than 10 were excluded from the analysis. Other causes of CLD were ruled out by clinical examination, imaging, biochemical tests, relevant serology, and histopathology of liver. Forty one patients underwent liver biopsy. Rest of patients were too sick to undergo liver biopsy, had deranged coagulation profile, low platelet count, severe hepatic dysfunction or refused to consent for liver biopsy. Interpretations of histological findings were performed using the Metavir scoring system.

HLA typing was performed to examine the correlation between the distribution of various HLA alleles and possible susceptibility for AIH. A total of 956 subjects (patients = 44, controls = 912) were tested for HLA A, B, and DR antigens. All the subjects in the control group were potential donors for renal transplantation. The control population was screened for any apparent liver disease which included clinical, laboratory and radiological workup. The HLA antigens A and B were tested by two-stage NIH microlymphocytotoxicity assay using eosin dye extrusion. Antisera trays were obtained from the collaborative transplant study (CTS) Heidelberg, Germany. HLA A and B were tested on two trays of AB1 and AB2 of 60 antisera each. Purified Tand B cells were used for Class I and Class II antigens by using monoclonal labeled magnetic beads (Dyna Beads). Relative allele frequencies were calculated via the total number of subjects. HLA DR antigens were determined by polymerase chain reaction (PCR) using sequence specific primers (SSP). Antisera trays and primers for HLA DR were obtained from the collaborative transplant study (CTS), Heidelberg, Germany. Clinical evaluation included history of alcohol and drugs, and physical examination. The laboratory workup included complete blood count, coagulation profile, liver function tests, serum urea, creatinine, electrolytes, and hepatitis B and C serological tests. Autoimmune serology comprised of antinuclear antibody (ANA), anti smooth muscle antibody (ASMA), Anti liver-kidney microsome antibodies (LKM), Anti soluble liver antigen antibodies (SLA), Anti mitochondrial antibodies (AMA), anti neutrophil cytoplasmic antibodies (p ANCA), and serum globulin levels. To exclude other underlying liver diseases, other tests including serum ceruloplasmin, ferritin, α1-anti trypsin levels, and fasting lipid profile were performed. Radiological evaluation was performed by abdomen ultrasonography and Doppler studies. Screening esophagogastroduodenoscopy (EGD) was performed when deemed necessary. Clinical and laboratory data were encoded and entered to ‘Statistical Package for Social Sciences’ software (SPSS, version 16.0). Continuous variables were expressed as median with ranges and categorical variables as absolute number with percentages. Data was analyzed by two-tailed Fisher exact test, and odds ratio derived from a 2 by 2 contingency tables. A P value of < 0.05 was considered as statistically significant.

## 4. Results

Of 58 cases, 35 were females (60.3%). Most patients were children and adolescents with the median age of 14 (range: 4-70 years). Demographical and clinical parameters are presented in Table 1. Clinical presentation was varied from asymptomatic derangement of liver function tests to acute hepatitis or decompensated liver disease. The presenting complains included generalized body weakness, joint pains, fever, jaundice, abdominal pain, and abdominal distension. Most had portal hypertension evident from hepatosplenomegaly with ascites. Cirrhosis, as determined clinically and radiologically, was present in 49 (84.4%) patients; Child class A in 13 (26.5%), Child B in 19 (38.7%), and Child C in 17 (34.6%). Decompensated cirrhosis with ascites was present in 24 (41.4%). Among 28 patients who underwent screening EGD, 20 (71.4%) had esophageal varices. 

**Table 1.** Characteristics of Patients With Autoimmune Hepatitis

	Data
**Gender, Male/Female, No.**	23/35
**Age, median (range), y**	14.5 (4-70)
**Presenting complaints, No. (%)**	
Fever	43 (74.1)
Abdominal pain	40 (68.9)
Jaundice	38 (69.7)
Abdominal distension	27 (46.5)
**Examination findings, No. (%)**	
Edema	09 (15.5)
Hepatomegaly	49 (84.4)
Splenomegaly	38 (66.7)
Ascites	24 (41.4)
Cirrhosis	49 (84.1)
Child A	13 (26.5)
Child B	19 (38.7)
Child C	17 (34.6)
Esophageal varices, (n = 28)	20/8 (71.4)
Decompensated disease	24 (41.3)
**Laboratory tests, median (range)**	
Total Bilirubin, mg/dL	2.1 (0.13-32.8)
Alanine aminotransferase, IU/L	74.5 (15-796)
Aspartate aminotransferase, IU/L	94 (12-1068)
Gamma-glutamyl transpeptidase, IU/L	56 (12-867)
Alkaline Phosphatase, IU/L	222 (54-1447)
International normalization ration	1.29 (0.87-3.1)
Albumin, g/dL	2.9 ( 1.1-4.7)
IgG levels, g/L	24.7 (10.2-55.6)
AIH score	14 (10-22)

Median International AIH Group scoring was 14 with a range of 10-22. Median laboratory tests values with ranges include: total bilirubin 2.1 mg/dL (0.13-32.8), alanine aminotransferase (ALT) 74.5 U/L (15-796), aspartate aminotransferase (AST) 94 U/L (12-1068), gamma-glutamyl transpeptidase (GGT) 56 U/L (12-867), alkaline phosphatase (ALP) 222 U/L (54-1447), International Normalized Ratio (INR) 1.29 (0.87-3.1), albumin 2.9 gr/dL (1.1-4.7), and IgG 24.7 gr/L (10.2-55.6).

### 4.1. Types of AIH

Thirty six (62%) patients had type1 AIH, 10 (17.2%) had type 2, and the remaining 12 were seronegative with biopsy proven AIH. Two of our patients who had negative results for ANA, ASMA, AMA serology, were found to have positive findings for Anti SLA.

### 4.2. Other Associated Diseases 

Of 15 patients who had associated diseases, overlap syndrome (according to Paris classification) (16), was seen in three patients (17), celiac disease coexisted in four (18), hyperthyroidism in one (19), diabetes mellitus in five (20), superior vena cava obstruction diagnosed in one (21), Sjogren’s syndrome in one (22), and nephrotic syndrome in one patient (23). One patient was found to have develop hepatocellular carcinoma incidentally (24), and underwent transarterial chemo-embolization (TACE).

### 4.3. Liver Biopsy Findings

**Figure 1.**
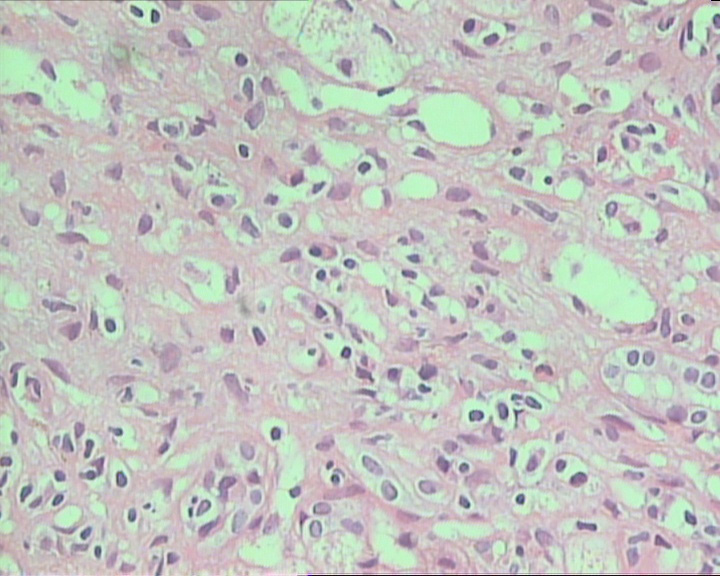
Photomicrograph Showing Expanded Portal Tract With Plasma Cell Rich Infiltration and Rosette Formation (H & E Stain X 400)

Among 41 patients who had biopsy proven AIH, features included interface hepatitis or piecemeal necrosis, plasma cell infiltration along with lobulitis. Four patients had hepatic rosette formation (Figure 1), and 16 of them had additional features consistent with cholestasis and bile ductular proliferation. Thirty-two patients had advance stages III and IV disease, and 20 had severe grade of inflammation. 

### 4.4. HLA Allele Frequencies

HLA A2 (P = 0.036), HLA A9 (23) (P = 0.018), HLA A10 (25) (P = 0.000), HLA A19 (33) (P = 0.000), HLA B15 (63) (P = 0.007), HLA B40 (61) (P = 0.002), HLA DR6 (P = 0.001) with its subtypes HLA-DRB1*13 (P = 0.032), and HLA-DRB1*14 (P = 0.017) were more prevalent in patients with AIH compared to controls (Table 2, 3, and 4). HLA DR 3 is prevalent in our normal population as well; so despite a more frequent allele, it was not statically significant. On the other hand HLA DR8 and DR9 were absent in our patients. 

**Table 2.** Distribution of HLA A Alleles in Patients With Autoimmune Hepatitis and Controls

HLA A	Patients’ Alleles = 88, (n = 44)	Controls Alleles = 1824, (n = 912)	Odds Ratio, (Confidence Interval)	P Value
**A1**	10	138	0.72 (0.34-1.48)	0.432
**A2**	10	190	0.49 (0.23-0.99)	0.036
**A3**	07	52	1.43 (0.57-3.40)	0.348
**A9**	10	94	1.12(0.52-2.32)	0.716
**A9 (23)**	03	04	8.01 (1.399-43.157)	0.018 ^a^
**A9 (24)**	07	90	0.79 (0.32-1.84)	0.707
**A10**	07	98	0.72 (0.30-1.67)	0.584
**A10 (25)**	04	00	∞ (6.96-∞)	0.000 ^a^
**A10 (26)**	03	85	0.34 (0.08-1.16)	0.074
**A10 (34)**	00	13	0.00 (0.00-4.12)	0.619
**A11**	19	134	1.60 (0.90-2.82)	0.089
**A19**	20	134	1.79 (0.97-2.99)	0.062
**A19 (29)**	02	18	1.16 (0.18-5.30)	0.693
**A19 (30)**	01	22	0.465(0.023-3.30)	0.717
**A19 (31)**	03	32	0.97 (0.23-3.40)	1.000
**A19 (32)**	01	24	0.43 (0.03-3.00)	0.718
**A19 (33)**	13	38	3.99 (1.92-8.15)	0.000 ^a^
**A28**	05	70	0.73 (0.25-1.93)	0.671
**Ax**	00	02	0.00 (0.00-42.63)	1.000

^a^ significant P values.

**Table 3.** Distribution of HLA B Alleles in Patients With Autoimmune Hepatitis and Controls

HLA B	Patients’ Alleles = 88, (n = 44)	Controls Alleles = 1824, (n = 912)	Odds Ratio, (Confidence Interval)	P value
**B 5**	12	124	1.00 (0.50-1.96)	1.000
**B5 (51)**	12	98	1.31 (0.65-2.59	0.376
**B5 (52)**	00	26	0.00 (0.00-1.95)	0.159
**B7**	03	36	0.86 (0.21-2.99)	1.000
**B8**	13	108	1.29 (0.66-2.49)	0.395
**B12**	04	64	0.63 (0.19-1.86)	0.507
**B12 (44)**	04	62	0.65 (0.20-1.92)	0.507
**B12 (45)**	00	02	0.00 (0.00-42.63)	1.000
**B13**	01	22	0.47 (0.02-3.30)	0.714
**B14**	00	04	0.00 (0.00-16.08)	1.000
**B15 **	07	50	1.49 (0.60-3.56)	0.333
**B15 (62)**	01	35	0.29 (0.01-1.99)	0.361
**B15 (63)**	05	10	5.43 (1.58-17.81)	0.007^a^
**B15 (70)**	01	05	2.09 (0.09-18.58)	0.425
**B16**	01	22	0.47 (0.02-3.30)	0.714
**B16 (38)**	01	14	0.74 (0.04-5.44)	1.000
**B16 (39)**	00	08	0.00 (0.00-7.06)	1.000
**B17**	10	85	1.25 (0.58-2.59)	0.566
**B17 (57)**	10	54	2.04 (0.93-4.34)	0.064
**B17 (58)**	00	31	0.00 (0.00-1.62)	0.103
**B18**	01	21	0.49 (0.02-3.47)	0.713
**B21 **	04	38	1.01 (0.32-3.31)	0.781
**B21 (49)**	01	07	1.49 (0.07-12.18)	0.523
**B21 (50)**	03	31	1.00-(0.24-3.53)	1.000
**B22**	03	33	0.94 (0.23-3.29)	1.000
**B22 (54)**	00	02	0.00 (0.00-42.63)	1.000
**B22 (55)**	03	31	1.00 (0.24-3.53)	1.000
**B27**	01	26	0.39 (0.02-2.75)	0.504
**B35**	12	110	1.15 (0.57-2.26)	0.612
**B37**	01	16	0.64 (0.03-4.69)	1.000
**B40**	14	118	1.27 (0.66-2.40)	0.412
**B40 (60)**	04	87	0.45 (0.14-1.32)	0.171
**B40 (61)**	10	31	3.64 (1.60-8.10)	0.002 ^a^
**B41**	00	02	0.00 (0.00-42.63)	1.000
**B42**	00	04	0.00 (0.00-16.08)	1.000
**B46**	00	02	0.00 (0.00-42.63)	1.000
**B47**	00	10	0.00 (0.00-5.50)	1.000
**B48**	00	02	0.00 (0.00-42.63)	1.000
**B53**	00	15	0.00 (0.00-3.53)	0.635

^a^ significant P values.

**Table 4.** Distribution of HLA DR Alleles in Patients With Autoimmune Hepatitis and Controls

HLA DR	Patients’ Alleles = 88, (n = 44)	Controls Alleles = 1824, (n = 912)	Odds Ratio, (Confidence Interval)	P Value
**DR1**	03	50	0.61 (0.15-2.08)	0.616
**DR2**	18	175	1.08 (0.61-1.92)	0.778
**DR2 (15)**	17	167	1.07 (0.59-1.92)	0.775
**DR2 (16)**	01	08	1.28 (0.06-10.22)	0.565
**DR3 (17) **	25	218	1.26 (0.75-2.11)	0.363
**DR4**	02	61	0.32 (0.05-1.38)	0.162
**DR5**	05	98	0.50 (0.17-1.32)	0.196
**DR5 (11)**	05	87	0.57 (0.20- 1.51)	0.332
**DR5 (12)**	00	11	0.00 (0.000-4.95)	0.612
**DR6**	28	154	2.30 (1.38-3.81)	0.001^a^
**DR6 (13)**	19	116	1.89 (1.06-3.35)	0.032
**DR6 (14)**	09	38	2.62 (1.13-5.89)	0.017^a^
**DR7**	05	102	0.48 (0.17-1.26)	0.146
**DR8**	0	22	0.00 (0.00-2.33)	0.249
**DR9**	0	18	0.00 (0.00-2.90)	0.394
**DR10**	02	14	1.49 (0.23-7.03)	0.645

^a^significant P values.

## 5. Discussion

Autoimmune hepatitis (AIH) is considered a rare disease in our part of world (25); It accounted for 0.03% of all patients visiting in our Hepatology clinic over the last ten years. Female dominance goes along with the published literature (10, 26). The clinical manifestations of AIH are broad with asymptomatic clinical course to severe acute illness (27, 28). These clinical presentations differ amongst racial factions. Afro-American patients present more with cirrhosis as compared to white Americans (29, 30). While natives of Alaska are diagnosed with acute AIH in comparison with nonnative inhabitants (31). Patients from the Middle East and Japan frequently have cholestatic pattern of LFT’s (32-34). Our patients had high prevalence of cirrhosis at the time of presentation. Though most were of pediatric age group, disease had already been very advanced. This trend is similar to what reported in South American children with AIH (35, 36). One third of our patients had decompensated liver disease. The most plausible reason for delay in the referral and diagnosis is lack of clinical suspicion of AIH as a cause of CLD (8). Type I AIH was more prevalent in our patients as compared to type II AIH which is congruent with the North American and the Western studies. However, percentage of this type is less as compared to other Asian countries (37). As already reported in international literature about the prevalence of seronegative AIH (38-40), twelve of our patients had this entity. The role of liver biopsy becomes of utmost importance in diagnosing this subset of patients (41).

Association of AIH with other autoimmune disorders has been described before (16). A significant number of patients in our study had associated autoimmune entities which included overlap syndrome, celiac disease, thyroid disorders, diabetes mellitus, superior vena cava obstruction, Sjogren’s syndrome, and nephrotic syndrome. One of our patients developed hepatocellular carcinoma as a rare etiology having AIH (24). Liver histology is supportive of AIH diagnosis as well as its usefulness in guiding the treatment decision. The salient and characteristic features include marked interface hepatitis or piecemeal necrosis, plasma cell infiltrates consistent with lobulitis, along with varying degree of fibrosis (41-45). Similar features were noted in all our patients who underwent liver biopsy. None of our patients had eosinophilic infiltrates or granuloma formation. However four patients showed changes of hepatic rosette formation (46, 47). Two of our overlap patients with positive results for AMA testing had histological findings of bile ductular proliferation and advanced fibrosis along with characteristic features of AIH (48-53). Those patients who had histopathological cholestasis had predominantly advanced liver fibrosis as well as high GGT levels as compared to those who had early stage of disease with normal GGT levels. The importance of genetic predisposition rendering an individual more vulnerable to have AIH has been comprehensively studied, and certain salient genetic relationships had been identified in past studies. HLA associations with AIH are diverse pertaining to different ethnic groups. The genetic backdrop in AIH has impact on disease severity, clinical presentation and treatment responses. The incongruity of the HLA genotypes found in various countries is not clearly explained. This variation is probably considered to be due to racial differences, ecological and geological factors or disease variation along with pattern of HLA alleles. HLA DR3 and HLA DR4 were more common in AIH Caucasian patients (54). Prevalence of DR3 in Japanese population was rarely found and AIH was associated with DR4; especially in older age group. Frequency of HLA-DR3 was increased in Italian and German patients (55-57). HLA DR13 has been seen consistently in Argentineans (58); Brazilian patients had HLA DR 3, DR13, and DR 7 as susceptibility alleles (59); Mexicans showed HLA DR 1 association with AIH (60). Previous local HLA frequencies in Pakistani population (61) showed HLA A11, HLA B 40, HLA DR2, and DR3 which were common in all ethnic groups including Sindhi, Punjabi, Urdu speaking, and pashtuns. This mixed picture shows similarities to both Caucasians and Orientals. In our study, HLA DR 2 and DR 3 were more prevalent in the control population. Why our normal population with prevalent HLA DR 2 and DR 3 alleles does not have AIH in significant number is an open question. Perhaps dietary and environmental factors play a protective role and decrease the susceptibility and vulnerability to develop AIH. Considering this ethnic diversity, determination of any association of HLA typing with AIH in our population becomes a difficult task. In spite of that we found HLA DR6 with its subtypes HLA-DRB1*13 and HLA-DRB1*14 more prevalent in patients with AIH compared to controls. Four of our patients were homozygous with DR6. Similar to our study, South American children with AIH have strong association with HLA DR6 and its subtype DRB1* 13 (36). Literature pertaining to the association of HLA A and HLA B antigens with AIH has not been extensively evaluated before. Their significance remains uncertain. We found association of HLA A2, HLA A9 (23), HLA A10 (25), HLA A19 (33), HLA B15 (63), and HLA B40 (61) in our patients with AIH in our study. However, we could not study the HLA DQ alleles. In conclusion, our patients of AIH presented with advanced disease, most belonged to pediatric and adolescent age group. One fifth of our patients had negative results for serology study, highlighting the need for liver biopsy for diagnosis. We identified genetic association of HLA DR 06 along with others in our patients with AIH.
